# Prediction of Mortality in Surgical Intensive Care Unit Patients Using Machine Learning Algorithms

**DOI:** 10.3389/fmed.2021.621861

**Published:** 2021-03-31

**Authors:** Kyongsik Yun, Jihoon Oh, Tae Ho Hong, Eun Young Kim

**Affiliations:** ^1^Computation and Neural Systems, California Institute of Technology, Pasadena, CA, United States; ^2^Department of Psychiatry, College of Medicine, Seoul St. Mary's Hospital, The Catholic University of Korea, Seoul, South Korea; ^3^Division of Hepato-Biliary and Pancreas Surgery, Department of Surgery, College of Medicine, Seoul St. Mary's Hospital, The Catholic University of Korea, Seoul, South Korea; ^4^Division of Trauma and Surgical Critical Care, Department of Surgery, College of Medicine, Seoul St. Mary's Hospital, The Catholic University of Korea, Seoul, South Korea

**Keywords:** anesthesia and intensive care, informatics, intensive care, surgery, machine learning

## Abstract

**Objective:** Predicting prognosis of in-hospital patients is critical. However, it is challenging to accurately predict the life and death of certain patients at certain period. To determine whether machine learning algorithms could predict in-hospital death of critically ill patients with considerable accuracy and identify factors contributing to the prediction power.

**Materials and Methods:** Using medical data of 1,384 patients admitted to the Surgical Intensive Care Unit (SICU) of our institution, we investigated whether machine learning algorithms could predict in-hospital death using demographic, laboratory, and other disease-related variables, and compared predictions using three different algorithmic methods. The outcome measurement was the incidence of unexpected postoperative mortality which was defined as mortality without pre-existing not-for-resuscitation order that occurred within 30 days of the surgery or within the same hospital stay as the surgery.

**Results:** Machine learning algorithms trained with 43 variables successfully classified dead and live patients with very high accuracy. Most notably, the decision tree showed the higher classification results (Area Under the Receiver Operating Curve, AUC = 0.96) than the neural network classifier (AUC = 0.80). Further analysis provided the insight that serum albumin concentration, total prenatal nutritional intake, and peak dose of dopamine drug played an important role in predicting the mortality of SICU patients.

**Conclusion:** Our results suggest that machine learning algorithms, especially the decision tree method, can provide information on structured and explainable decision flow and accurately predict hospital mortality in SICU hospitalized patients.

## Introduction

Prediction of mortality rate of patients in intensive care unit (ICU) has been a critical issue (1–3).To assess the probability of death in ICU patients, several models using routine admission variables (4) and objectively derived weights were proposed in the 1980s (5). Along with these attempts, Acute Physiology And Chronic Health Evaluation (APACHE) II was developed to assess the severity and mortality of patients admitted to ICU in 1985 (6, 7). Other scoring systems such as Simplified Acute Physiology Score (SAPS) II that can provide a probability of hospital mortality have also been suggested (8). With new variables such as Glasgow Coma Scale and thrombolysis, APACHE was updated to APACHE IV in 2006, showing better performance in predicting mortality rate in ICU patients (9). SAPS III also added several variables that could be quickly measured at admission, showing increased prediction performance compared to SAPS II (10).

However, they have several limitations in clinical settings although APAHCE, SAPS, and other scoring systems are widely used. First, as these prediction models only use a few variables, more precise and accurate prediction is difficult. SAPS III applies only 20 variables while APACHE IV uses 26 ones. This simplicity makes it possible to quickly determine the status of patients admitted to ICU (11). Second, SAPS and APACHE IV only assess physiological states of patients on the first day of admission. Although there are other scoring systems that can repetitively measure patients' status (e.g., Sequential Organ Failure Assessment named SOFA), the prognosis and mortality could not be accurately predicted from the data measured only once at the time of admission.

For these reasons, there have been several attempts to predict the mortality rate of critically ill patients using machine learning techniques. Support vector analysis could discriminate mortality in patients with hematologic malignancies (12). Random forest model can well-predict death from in-hospital patients, showing higher accuracy rate than Modified Early Warning Scores (MEWS) (13). Latent variable models that use information from electronic healthcare records predicted in-hospital death with combined time-varying model yielding the best performance (14) and it can also accurately estimate the probability of death in 1-year for multi-condition hospitalized patients (15). These findings demonstrate that the accuracy of mortality prediction for critically ill patients can be increased when machine learning algorithms and various medical data are used. However, it is currently unknown how machine learning algorithms make decisions during the prediction process. Thus, the objective of this study was to determine whether machine learning algorithms using demographic, laboratory, and other disease-related variables could predict in-hospital death of critically ill patients who were admitted to surgical intensive care unit (SICU) with considerable accuracy and identify factors contributing to the prediction power.

## Methods

### Participants Selection

**Figure 1:**
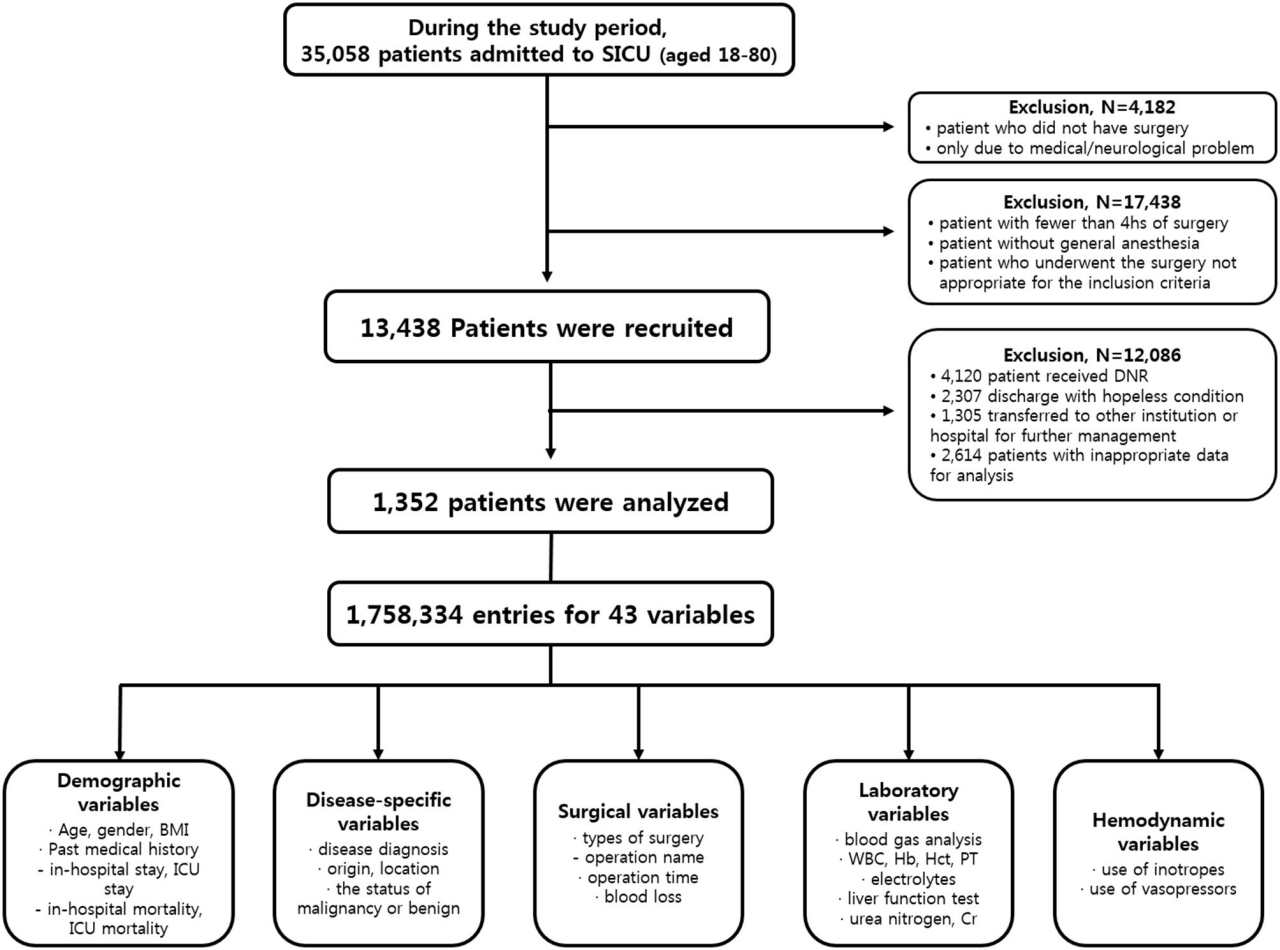
Flow diagram of participant selection.

From January 1990 to March 2017, patients admitted to SICU of our institution for postoperative management after major abdominal surgeries were included in this study. Our institution is a tertiary referral hospital and SICU has an average of 1,800 patients annually. Major abdominal surgeries were defined as operation under general anesthesia status with endotracheal tube over 4 h regardless of the type of diagnosis, the status of malignancy or benign, the type of surgery or surgical sites. Subjects who met any of the following features were excluded from study; (a) age <18 or >80 years, (b) the duration of SICU stay <24 h, (c) patient was admitted to SICU due to medical or neurological problem without operation, (d) hopeless condition of patient in medical aspects, (e) pregnant state, or (f) measurements required for our predictor were not recorded at any time during ICU stay. Finally, a total of 1,352 patients were enrolled for further analysis (Figure 1).

### Data Extraction

Clinical data and medical records during the study period were retrospectively reviewed. Authors used patient-level information and medical records extracted from electrical medical records (EMR) of our institution. Disease characteristics included the diagnosis of disease, origin or location of lesion, malignancy or benign status. The policy of vital sign measurement in our institution was prescribed to mandate the frequency of vital sign measurement to be two every hour unless otherwise specified. Variables of laboratory tests included results of arterial blood gas analysis and serum blood chemistry test. The usage of inotropes or vasopressors was also reviewed. The outcome measurement was the incidence of unexpected postoperative mortality defined as mortality without pre-existing not-for-resuscitation order that occurred within 30 days of the surgery or within the same hospital stay as the surgery. Finally, a total of 43 variables composed of 1,758,334 entries of enrolled patients were used for analysis. Detailed protocol of data extraction is presented in Figure 1. This study was approved by the Institutional Review Board of the Ethics Committee of our institution (IRB No. KC17RESI0672).

### Model Development and Validation

#### Decision Trees

Decision trees can predict classifications (life or death) from medical data and these have the advantage of being able to present decisions visually and explicitly (16). We can use the final decision tree to accurately explain why a particular prediction is performed. To predict the classification, the algorithm followed the tree's decision from the root (start) node to the leaf node (final classification). Each step of the prediction involved checking the value of one predictor variable. If predictor *x1* exceeded a certain value *n*, it would follow the right branch representing *type 1* (life). Otherwise, it would follow the left branch to indicate *type 0* (death). The purpose of training the decision tree was to create a model that could predict the value of target variable based on multiple input variables. We tried and tested multiple decision points to numerically sort all values using a greedy approach and to maximize the prediction performance of the target value. All input variables and decision points were evaluated and selected in a greedy manner based on cost function.

Tree partitioning continued until the node contained a minimum number of training examples or reached the maximum tree depth. The Gini cost function was used to indicate how good a decision split was, depending on how many classes were mixed in the two groups generated by the decision split (17). Data were divided into three sets; (1) 70% were used for training, (2) 15% were used for validating that the network was generalizing with training stopped before overfitting, and (3) 15% were used for completely independent test for network generalization. Moreover, 10-fold cross validation was used to test the stability of results by randomly shuffling training/validation/testing data sets.

#### Neural Networks

**Figure 2:**
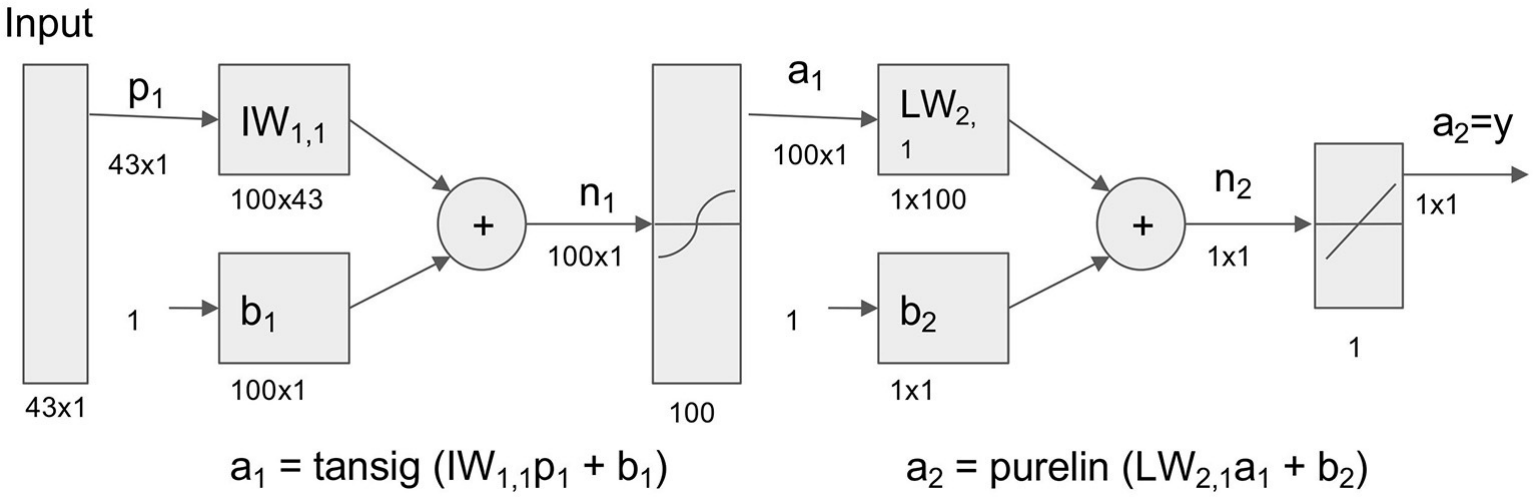
Neural network model for prediction of life and death in hospital. Neural network model to transform 43 Surgical Intensive Care Unit (SICU) medical data for prediction of life and death. Tansig = hyperbolic tangent sigmoid function; Purelin = linear transfer function.

Base model architecture is as follows (Figure 2). In this study, 43 variables were used as input variable to the neural network that consisted of one hidden layer with 100 neurons (parameters). A linear output neuron was used to obtain the final output of the regressive model. It is known that the model can fit multi-dimensional mapping problems arbitrarily well if consistent data are given with enough neurons in its hidden layer (18, 19).

**Figure 3:**
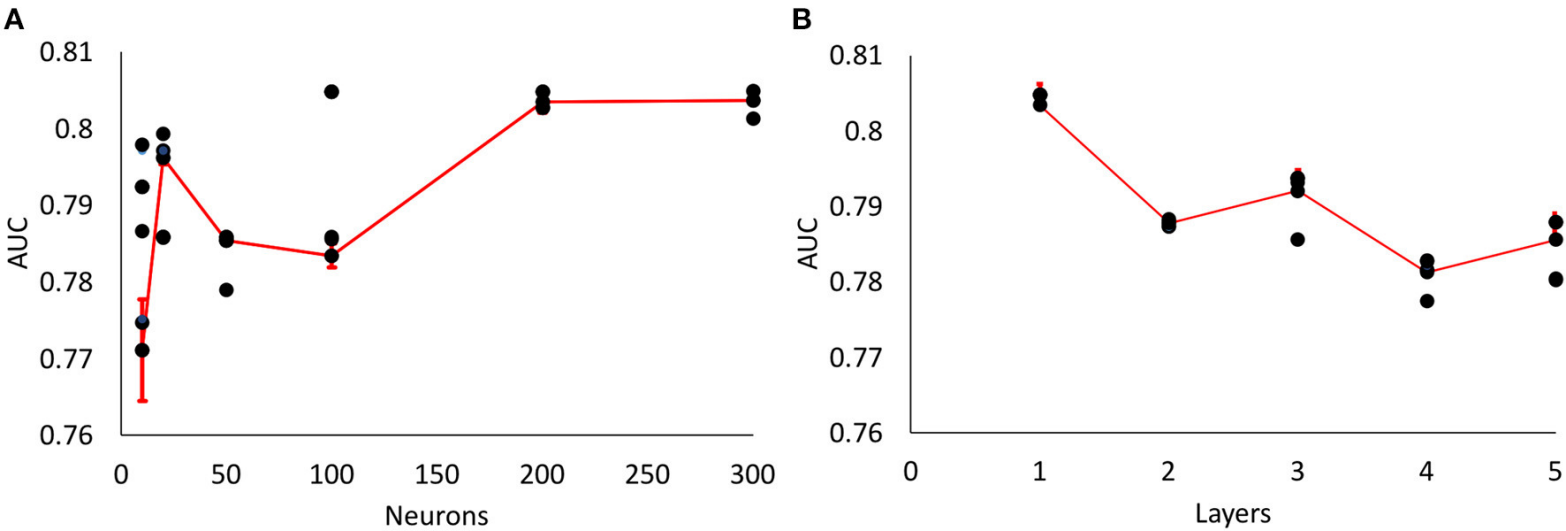
Parameter optimization for neural networks. **(A)** Area Under Curve (AUC) values from different number of neurons (single layer, 10, 20, 50, 100, 200, 300 neurons) (10-fold cross validation results). Maximum AUC at 200 neurons or more. **(B)** AUC values from different number of layers with 200 neurons (10-fold cross validation results). Maximum AUC at single layer (Error bars represent standard errors).

We tested different number of hidden neurons (10, 20, 50, 100, 200, 300 neurons) and layers (1–5 layers), and compared the performance (Figure 3). We found that the area under curve (AUC) is saturated at the number of neurons of 200 or more (10-cross validations), and the AUC is maximum at the single layer with 200 neurons. Therefore, we determined 200 hidden neurons (number of parameters) and a single layer for our neural network parameters.

The network was trained with scaled conjugate gradient backpropagation algorithm (20, 21). Same training (70%), validation (15%), and testing (15%) division as decision tree was used in neural networks. Ten-fold cross validation was also used, and we compared the results with decision tree using independent *t*-test (Matlab, MathWorks Inc.).

#### Naive Bayes

Naive Bayes is a classification algorithm that applies a density estimate to the data and assumes that the predicted variables are conditionally independent. Naive Bayes classifiers are known to produce a posterior distribution that is robust to biased class density estimates (22). The Naive Bayes classifier assigns observations to the most likely class (i.e., maximum post-decision rule). The algorithm first estimates the density of predicted variables within each class. It then models the posterior probability according to the Bayes rule. That is, for all *k* = *1, ..., K*,

(1) $$\begin{array}{r} \hat{P}\left(Y=k|X_{1},\ldots ,X_{p}\right)=\frac{\pi \left(Y=k\right)\prod_{j=1}^{P}P\left(X_{j}|Y=k\right)}{\sum_{k=1}^{K}\pi \left(Y=k\right)\prod_{j=1}^{P}P\left(X_{j}|Y=k\right)} \end{array}$$

Where *Y* is a random variable corresponding to the class index of the observation. *X*_1_*, ..., X*_*P*_ are random predictors of observation. π*(Y* = *k)* is a prior probability with class index *k*. The algorithm then classifies observations by estimating posterior probabilities for each class and assigning observations to classes that yield maximum posterior probabilities.

#### Random Forests and Hellinger Distance Estimates

Furthermore, to establish the model stability of imbalanced dataset (only 10% of participants belonged to expired class), we applied the Hellinger Distance Decision Tree (23), the Hellinger Distance Random Forest (24) and the Random Forest model (25). To test the machine learning model stability, we performed a 10-fold cross-validation and tested whether the machine learning model performance was significantly different depending on the various data selections for training and testing. Since the F1 score is the harmonic mean of precision and recall, statistical tests were only performed on the AUC and F1 scores.

## Results

### Participants and Variable Selection

The criteria used for patient selection and lists of variables are presented in Figure 1. During the inclusion period, 35,058 patients were admitted to SICU. Among them, 4,182 were excluded as they had medical or neurological problem without operation. Then 17,438 patients who underwent surgery for <4 h were excluded. Of 13,438 patients who were recruited after meeting the selection criteria, 12,086 patients with a “do not resuscitate form” or were discharged with hopeless condition were excluded. Analysis in more detail, among 12,086 patients, 4,120 patients received actual “do not resuscitate form,” 2,307 patients with discharge with hopeless condition, and 1,305 patients were transferred to other institution or hospital for further management. Additionally, there were 2,614 patients who were excluded from the analysis due to insufficient medical data. Thus, data of 1,352 patients were used for training machine learning algorithms. Forty-three variables consisting of demographic, laboratory, hemodynamic, surgical, and disease-specific variables were used to estimate mortality of SICU patients. Comparative analysis results of participants are presented in Table 1.

**Table 1:** Comparative analysis of demographics of enrolled patients according to the survival or expire.

**Variables**	Survivor^*^ (%)	Expired^**^ (%)	***p-value***
Number of patients	1,232 (91.1)	120 (8.9)	
Mean age (year)	50.6 ± 9.4	68.8 ± 10.3	0.023
Male/Female	848/384	82/38	0.918
Diagnosis			0.015
Malignancy	1,102 (89.4)	97 (80.8)	0.036
Upper GI tract	141 (11.4)	8 (6.7)	
Lower GI tract	293 (23.8)	24 (7.6)	
Hepatobiliary-pancreas	663 (53.8)	64 (8.8)	
Miscellaneous	5 (0.4)	1 (0.8)	
Benign	130 (10.6)	23 (19.2)	0.008
Hemoperitoneum	32 (2.6)	2 (1.7)	
Panperitonitis	87 (7.1)	8 (15)	
Biliary shock	5 (0.4)	0	
Miscellaneous	6 (0.5)	3 (2.5)	
Type of surgery			<0.001
Elective operation	1,020 (82.8)	78 (65)	
Emergency operation	212 (17.2)	42 (35)	

* *The patient survived more than 30 days after surgery or during the same hospital stay as the surgery*.

** *The unexpected postoperative mortality which was defined as the mortality without the pre-existing not-for-resuscitation order and occurred within 30 days of the surgery or within the same hospital stay as the surgery*.

### Prediction Performance of Mortality Using Machine Learning Algorithms

**Figure 4:**
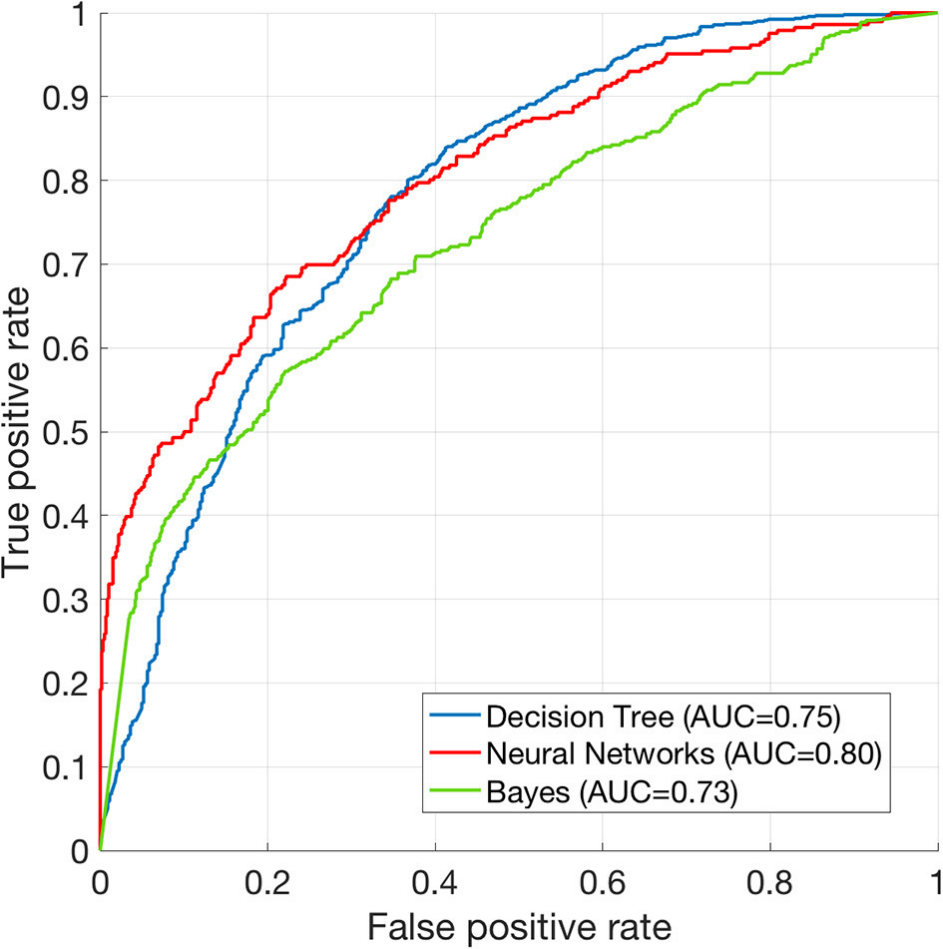
Receiver operating characteristics curve of machine learning algorithms. ROC curve of Decision tree (AUC = 0.75), neural net (AUC = 0.80), and Bayes (AUC = 0.73) classification algorithms. The results are based on 10-fold cross validations.

**Figure 5:**
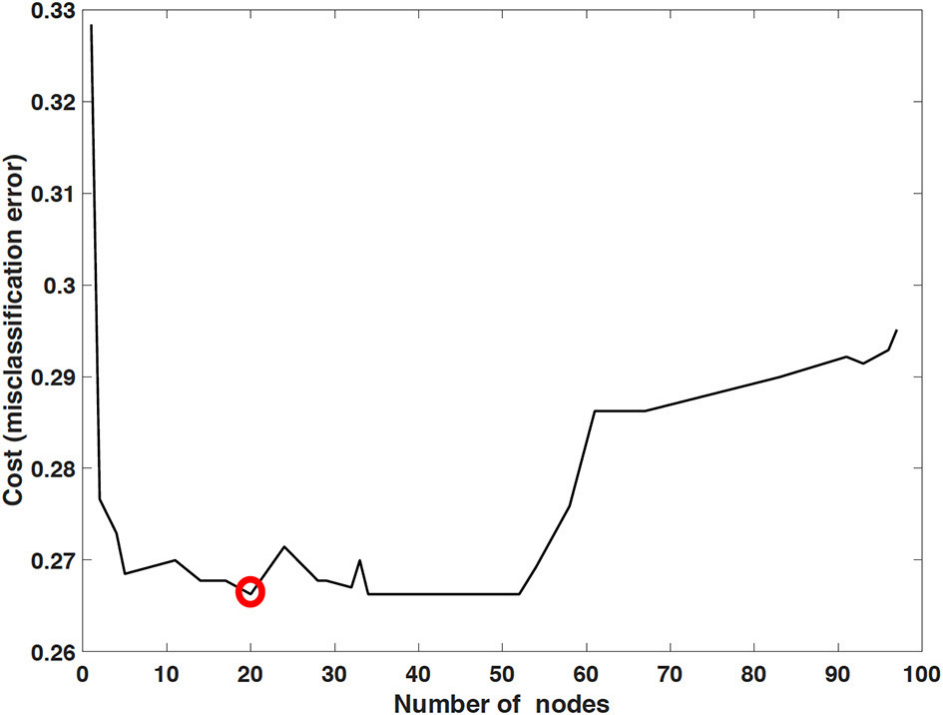
Optimized decision tree for the classification of life/death of patients in surgical intensive care unit.

The performance of mortality prediction is presented in Figure 4. Among decision tree, neural network, and Bayes classifier algorithms, the neural network algorithm showed the highest performance with an AUC of 0.80, followed by the decision tree with an AUC of 0.75. Bayes classifier had the least predictive accuracy, with an AUC of 0.73. As the decision tree algorithm has nodes that represent variables and conjunction that connects the nodes, the performance of this algorithm mainly depends on the number of nodes and tree size (26). Thus, we explored different ways to find the optimal performance of the decision tree algorithm by adjusting the number of nodes (Figure 5). We found that the optimal number of nodes that could minimize the decision tree's misclassification error rate was 77, where the classification prediction error was 0.2478 (75% classification accuracy). Using this number of nodes, decision tree structure was pruned. The results were based on the 10-fold cross validation.

We compared the 10-fold cross validation results between the neural networks and the decision tree algorithms. The AUCs among 10 validation runs were stable in that the standard deviation was 0.0012 for the decision tree, and 0.0017 for the neural networks. The independent *t*-test showed that t_(18)_ = 68.05 and *p* < 0.00001. Therefore, the neural network algorithm performed significantly better than decision tree.

To test whether the difference in F1 scores was significant in different machine learning models, we used the Kruskal-Wallis test, ANOVA's non-parametric counterpart. The results showed significant differences in the F1 score (Kruskal-Wallis chi square = 52.93, df = 5, *p* < 0.001). Then we tested the pairwise comparison using the Wilcoxon rank test between different ML models. Although Random Forest had the highest F1 score, we found no significant difference between Random Forest, Bayes, Decision Tree, and Neural Network ML models (*p* > 0.05). Compared to the random forest, the Hellinger distance decision tree and the Hellinger distance random forest showed a significant decrease in the F1 score (*p* < 0.001). When considering multiple comparison corrections, the significance level should be adjusted to 0.0017 (0.05/30 comparison) instead of 0.05.

The Kruskal-Wallis test showed significant differences in AUC values among various machine learning models (Kruskal-Wallis chi square = 43.75, df = 5, *p* < 0.001). We also tested pairwise comparisons using the Wilcoxon rank test and found no significant differences between the Random Forest, Bayes, Decision Tree, Neural Network, and Hellinger Distance Random Forest ML models (*p* > 0.05) (Table 2). Compared to the neural network model, the Hellinger distance decision tree showed a significant reduction in the AUC value (*p* < 0.001).

**Table 2:** Performance metrics of each ML model (Wilcoxon rank test, *p < 0.0017, adjusted *p*-value for multiple comparisons).

	**F1 score**	**Precision**	**Recall**	**AUC**
Decision tree	0.72	0.62	0.87	0.75
Neural networks	0.83	0.74	0.95	**0.80**
Bayes	0.82	0.70	**0.98**	0.73
Random forest	**0.84**	**0.78**	0.90	0.77
Hellinger distance decision tree	0.48*	0.49	0.48	0.65*
Hellinger distance random forest	0.51*	0.51	0.51	0.74

*Bold value denote the highest value in each metric*.

### Optimized Decision Tree for the Classification of Life/Death

**Figure 6:**
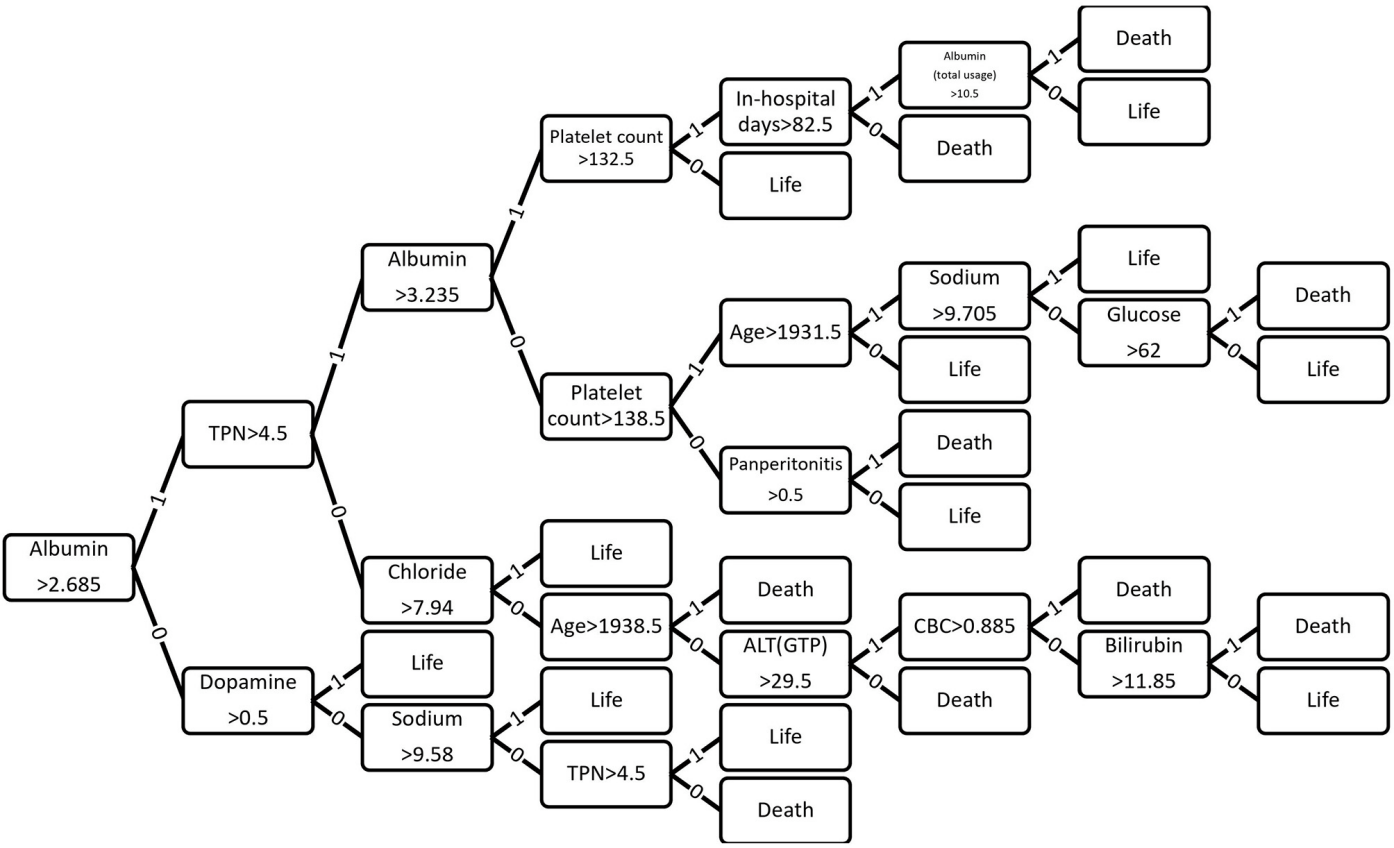
Contribution of 43 variables in predicting life or death of ICU patients (32, 33).

Figure 6 shows how 43 variables are applied to predict life or death of ICU patients. Among 43 variables, serum level of albumin had a crucial role in the prediction of mortality. If albumin level was higher than 2.685 g/dL, the number of days of total parental nutrition played an important role in the next decision. If albumin level was not higher than 2.685 g/dL, the peak dose of dopamine drug was important. If patient's albumin level was higher than 2.685 g/dL and the peak dose of dopamine level was higher than 8.3 mcg/kg/min, he/she was more likely to survive.

## Discussion

Herein, we showed that a certain machine learning algorithm could predict death of SICU patients using variables frequently used in clinical practice. Decision tree algorithm had a higher classification performance (AUC = 0.96) than neural network or Bayes classifier algorithm. This result might be applicable to clinical application considering results of other fields (27).

Previous studies have shown that machine learning algorithms could be used to predicting the prognosis and death of ICU patients. Both support vector machine and random forest model had an acceptable performance in predicting deaths of critically ill patients. Results of the present study showed somewhat higher performance than those of previous studies. It might be related to the number of variables used in training machine learning algorithms. In Verplancke's study, 12–17 variables were included in discriminating life and death of critically ill patients. However, our model used 43 variables (12). A small number of variables can be advantageous in helping clinician to make quick decisions as they do not require additional laboratory testing. However, since the accuracy of machine learning is related to the number of variables used, it may be more effective to use as many variables as possible to increase the prediction accuracy for mortality.

According to a recent observational cohort study comparing the performance of several machine learning algorithms using the same dataset, machine learning algorithms out-performed conventional scoring systems (e.g., MEWS) (13). In that study, random forest model had the highest performance (AUC = 0.80) whereas decision tree showed the lowest value (AUC = 0.73). Churpek et al. have also shown that basic physiological data (e.g., respiratory rate and heart rate) are the most significant predictors of deterioration of in-patients (13). These results were somewhat different from our results as laboratory test played a crucial role in our findings (Figure 4). This difference might be due to difference between machine learning algorithm used and outcome measurement used in different studies. While Churpek et al. focused on the deterioration of condition of in-patients, we aimed to discriminate life and deaths of SICU patients. Furthermore, we did not include basic physiological data when training machine learning algorithms to match time-resolution with other laboratory variables (laboratory tests were acquired every few days while heart rate and respiratory rates were acquired continuously in SICU). Acquisition of continuous data can inevitably lead to drawbacks of the data. Removal of electrocardiogram leads due to patient's movement can cause sustained zero heart rate which is the case of “false alarm” while under-sampling or erroneous data due to sensor fault can occur during care of ICU patients. These imprecise and missing data corruptions are primary challenges in critical care and it is still difficult to detect and correct these errors in large amounts of patient data (28). Therefore, this study included only objective variables that could be periodically measured. Thus, the present study could not confirm how physiological indicators contributed to mortality prediction. For this reason, it is difficult to directly compare results of this study with existing scoring systems (e.g., APACHE, MEWS, etc.). However, it can be compared with AUC performance reported in previous studies. APACHE II and SOFA showed AUC values of 0.81 and 0.71, respectively, in predicting prognosis in patients with ventilator-assisted pneumonia (29). MEWS and modified Mortality in Emergency Department Sepsis scores had AUC values of 0.61 and 0.77, respectively, in predicting 28-days mortality of patients in emergency department (30). Although characteristic of patients and the number of data are different, our findings suggest that mortality prediction using machine learning algorithms may have higher prediction accuracy than these classical scoring systems.

In results of optimized decision tree method, the most important and contributing variable in predicting mortality of SICU patients was albumin (Figure 4). Reduced level of serum albumin is known to be an independent predictor of mortality. In a large epidemiologic study, decrement of 2.5 g/L serum albumin is associated with increased odds of deaths (31). Preoperative serum albumin concentration also well-predicted operative mortality and morbidity (32, 33). Although serum albumin concentration was an important variable for predicting the prognosis and mortality of surgical patients in previous studies, we did not give any indication of its significance while training the machine learning algorithm. Nonetheless, decision tree algorithm identified that serum albumin concentration was the most important indicator for decision-making of life and deaths. This result suggests that machine learning algorithms might be able to recognize clinically significant factors in large data sets.

This study has several limitations. First, as mentioned above, physiological indicators such as heart rate or respiratory rate were not used for prediction. This makes it difficult to compare findings of our study with classical scoring indicators. Second, as this study used dataset of a single institution, it was impossible to compare differences in various patient groups or treatment protocols. Moreover, a large number of patients enrolled were excluded from the final analysis due to the lack of essential data. But this is due to the fact that the patients who had missing these parameters were strictly excluded from the analysis to ensure a high accuracy of the model and to confirm a strong correlation with the parameters, even if the representativeness of the whole group is somewhat less. An external validation via multicenter, prospective designed study should be conducted to confirm our results in the near future. Finally, only some variables in the electronic health record were used to train the machine learning algorithm. Thus, it may be necessary to include real-time variable data to improve the accuracy for mortality prediction of critically-ill patients.

In conclusion, our results suggest that machine learning algorithms, especially the decision tree method, can provide information on structured and explainable decision flow and accurately predict hospital mortality in SICU hospitalized patients.

## Data Availability Statement

The data analyzed in this study is subject to the restrictions. Data can be shared with permission from the institutional review board of the Catholic University of Korea. Requests to access these datasets should be directed to Eun Young Kim, freesshs@naver.com.

## Ethics Statement

The studies involving human participants were reviewed and approved by The Catholic University of Korea. Written informed consent for participation was not required for this study in accordance with the national legislation and the institutional requirements.

## Author Contributions

KY and EK build research design and collected. KY, JO, and TH analyzed and interpreted the patient data. JO and KY were a major contributor in writing the manuscript. All authors read and approved the final manuscript.

## Conflict of Interest

The authors declare that the research was conducted in the absence of any commercial or financial relationships that could be construed as a potential conflict of interest.

## Abbreviations

ICU: Intensive care unit
APACHE: Acute physiology and chronic health evaluation
SAPS: Simplified acute physiology score
AUC: Area under the receiver operating characteristic curve
SICU: Surgical intensive care unit
EMR: Electrical medical records
AST: Aspartate transaminase
ALT: Alanine transaminase
MEWS: Modified early warning score
SOFA: Sequential organ failure assessment.
