# Supplementary figures and images for: Prediction of Mortality in Surgical Intensive Care Unit Patients Using Machine Learning Algorithms
Source: Front Med (Lausanne). 2021 Mar 31;8:621861. doi: 10.3389/fmed.2021.621861 (PMC8044535; doi:10.3389/fmed.2021.621861)

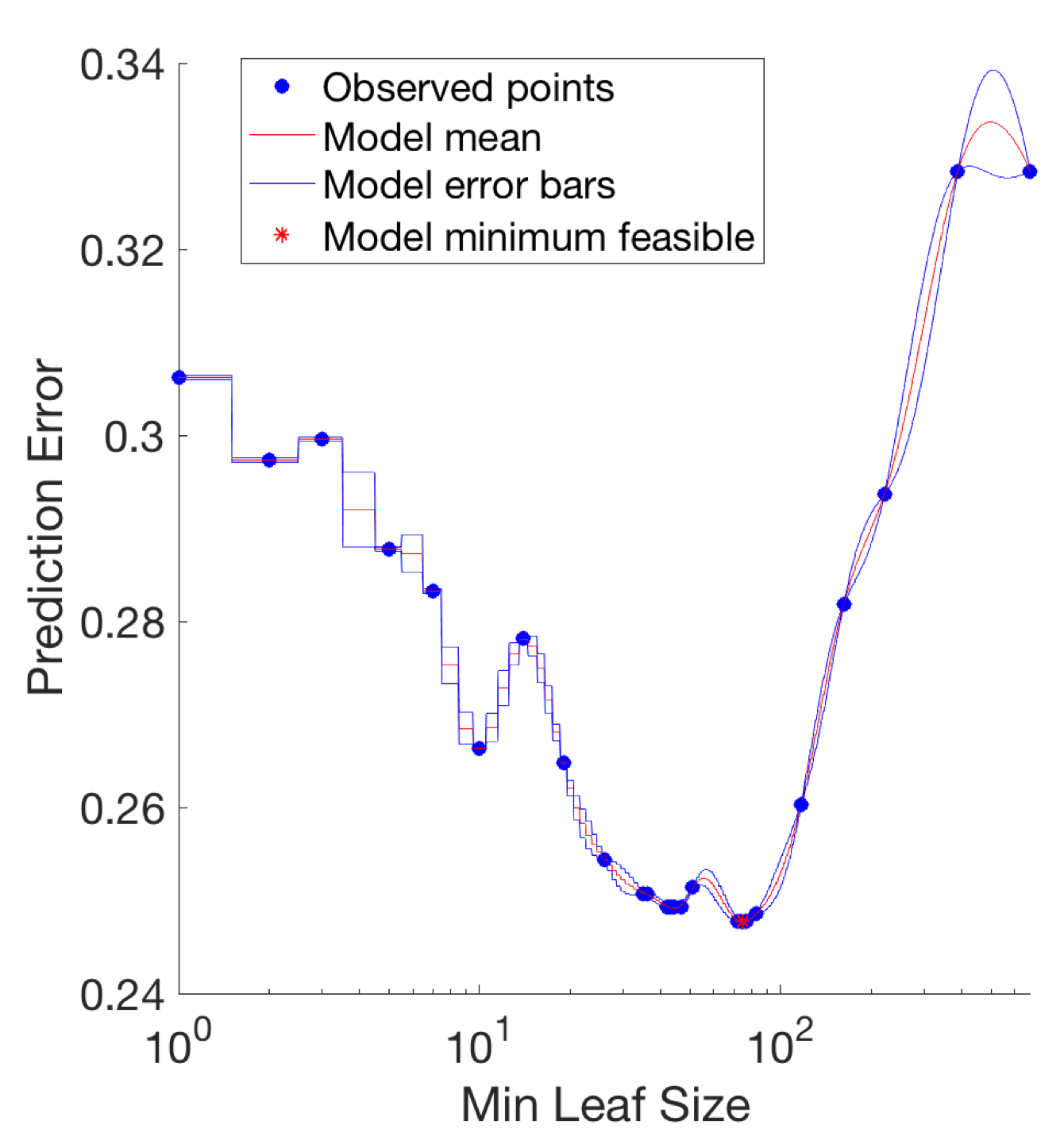

Supplement: Supplementary file 1 [file Image_1.PNG]
